# Simultaneous Determination of Multiple Components in Guanjiekang in Rat Plasma via the UPLC–MS/MS Method and Its Application in Pharmacokinetic Study

**DOI:** 10.3390/molecules21121732

**Published:** 2016-12-16

**Authors:** Jian Wu, Ying Xie, Zheng Xiang, Canjian Wang, Hua Zhou, Liang Liu

**Affiliations:** 1 State Key Laboratory of Quality Research in Chinese Medicine (Macau University of Science and Technology), Taipa, Macau, China; wujian071@163.com (J.W.); yxie@must.edu.mo (Y.X.); rainbowaftersnow@163.com (Z.X.) wangcanjianabc@163.com (C.W.); 2 Faculty of Chinese Medicine, Macau University of Science and Technology, Taipa, Macau, China

**Keywords:** Guanjiekang (GJK), pharmacokinetic study, UPLC–MS/MS method, simultaneous determination of multi-components

## Abstract

Guanjiekang (GJK) that is formed by five medicinal herbs including *Astragali Radix*, *Aconiti Lateralis Radix Praeparaia*, *Glycyrrhizae Radix et Rhizoma, Corydalis Rhizoma* and *Paeoniae Radix Alba* was used for the treatment of rheumatoid arthritis (RA). However, the pharmacokinetic (PK) profile of active components in GJK remains unclear. This study aims to evaluate the pharmacokinetic behavior of seven representative active constituents in GJK (i.e., benzoylhypaconine, benzoylmesaconine, paeoniflorin, tetrahydropalmatine, calycosin-7-glucoside, formononetin and isoliquiritigenin) after oral administration of GJK in rats. A rapid, sensitive and reliable ultra-performance liquid chromatography-tandem mass spectrometer (UPLC–MS/MS) method has been successfully developed for the simultaneous determination of these seven constituents in rat plasma. Chromatographic separation was achieved on a C_18_ column with a gradient elution program that consists of acetonitrile and water (containing 0.1% formic acid) at a flow rate of 0.35 mL/min. Detection was performed under the multiple reaction monitoring (MRM) in the positive electrospray ionization (ESI) mode. The calibration curves exhibited good linearity (R^2^ > 0.99) over a wide concentration range for all constituents. The accuracies ranged from 92.9% to 107.8%, and the intra-day and inter-day precisions at three different levels were below 15%. Our PK results showed that these seven compounds were quickly absorbed after the administration of the GJK product, and T_max_ ranged from 30 min to 189 min. The in vivo concentrations of paeoniflorin and isoliquiritigenin were significantly higher than the reported in vitro effective doses, indicating that they could partly contribute to the therapeutic effect of GJK. Therefore, we conclude that pharmacokinetic studies of representative bioactive chemicals after administration of complex herbal products are not only necessary but also feasible. Moreover, these seven compounds that were absorbed in vivo can be used as indicator standards for quality control and for determining pharmacokinetic behavior of herbal medicines in clinical studies.

## 1. Introduction

The constituents in Chinese herbal medicine (CHM) are complex and diverse. These constituents are the material basis for producing the therapeutic efficacy of CHM in the clinic. The effects of CHM are mediated via multi-target and multi-pathway actions of multiple components during the preparation of CHM [1,2]. Moreover, these multiple components are believed to generate a synergistic effect for treating illnesses, rather than the pharmacological actions produced by a single compound [3,4,5,6]. Therefore, the pharmacokinetic (PK) studies on multiple components are more valuable for elucidating the pharmacological mechanisms for treating relevant diseases as well as for ensuring the quality of CHM preparations [7,8,9,10]. However, the method of simultaneous determination of multiple constituents in rat plasma for the pharmacokinetic study of CHM preparations is significantly more difficult than the method for determining the PK behavior of a single chemical compound.

Guanjiekang (GJK) is an herbal medicinal preparation for treating rheumatoid arthritis (RA). It is derived from a classic Chinese herbal formula named Wutou Decoction, which has been used for thousands of years and is recorded in the “Synopsis of Golden Chamber”, which was written by the famous Chinese medical doctor Zhongjing Zhang during the Han Dynasty in China. The composition of GJK includes *Astragali Radix*, *Aconiti Lateralis Radix Praeparaia*, *Glycyrrhizae Radix et Rhizoma, Corydalis Rhizoma* and *Paeoniae Radix Alba.* Previous studies showed that the five herbs in the GJK preparation had significant suppressive effects on the arthritic and acute inflammatory animal models [11,12,13]. In our preliminary studies, the systemic quality control system has been established for GJK [14] that, together with the optimal manufacturing process and the significant effects of anti-arthritis and antinociception in rats, consequently confirms the safety and effectiveness of GJK. However, whether these constituents can reach the effective concentrations in plasma and manifest a rational PK behavior to have the therapeutic effects remains unknown [15,16]. Moreover, there may be synergistic effects for treating RA via positive herb–herb and compound–compound interactions in GJK. All of these questions need further in vivo PK studies to provide answers.

In a previous study, a reliable method using ultra performance liquid chromatography–tandem mass spectrometer (UPLC–MS/MS) was established for a simultaneous quantitative analysis of 13 major constituents in the pharmaceutical preparation of GJK for quality control [14]. However, considering the complexity of the matrix effect in biological samples and trace amount of these active compounds in vivo, it is still a significant challenge for the simultaneous determination of these active compounds in plasma after the administration of herbal preparation. In the current study, our goal was to establish a sensitive and reliable method using UPLC–MS/MS analysis for the simultaneous determination of seven constituents in rat plasma and to apply this method to the pharmacokinetic study of GJK preparation. These seven constituents including benzoylmesaconine, benzoylhypaconine [17,18], paeoniflorin [19], tetrahydropalmatine [20,21], calycosin-7-glucoside [22], formononetin [23], and isoliquiritigenin [24,25] have been considered as represented active compounds for five herbs and used as indicators of quality control and of determining pharmacokinetic behavior of five herbs or Proprietary Chinese Medicines containing them. The PK profiles of these constituents after oral administration of GJK in rats will be helpful for evaluating the safety and effectiveness of herbal medicinal preparation.

## 2. Results and Discussion

### 2.1. Optimization of Chromatographic Conditions

**Figure 1:**
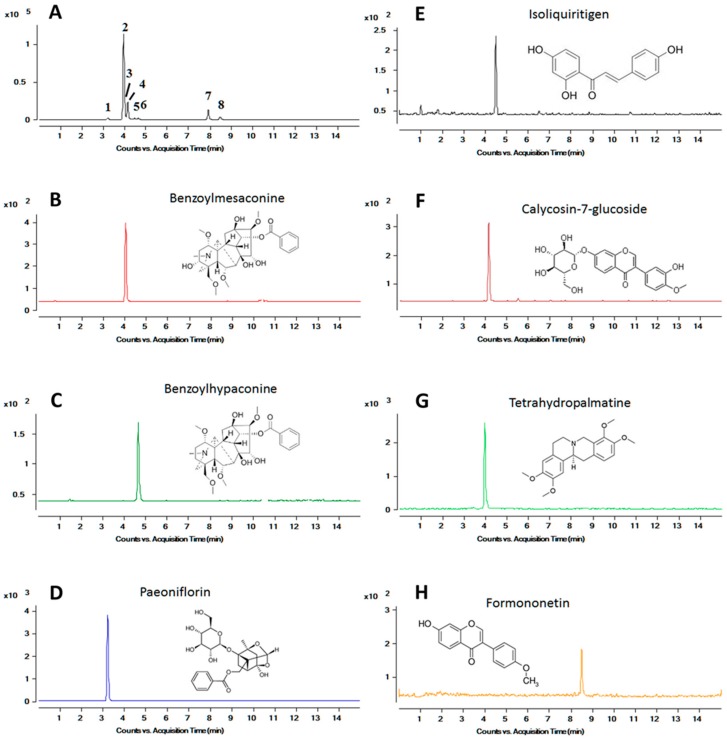
Representative UPLC-MS chromatograms for simultaneous determination of seven compounds and naringenin (IS) in rat plasma. (**A**) Representative chromatograms of the blank plasma sample spiked with seven analytes and IS. (1) paeoniflorin; (2) tetrahydropalmatine; (3) benzoylmesaconine; (4) calycosin-7-glucoside; (5) benzoylhypaconine; (6) isoliquiritigenin; (7) IS; and (8) formononetin. Representative chromatograms of the rat plasma sample obtained 1 h after the oral administration of GJK with (**B**) benzoylmesaconine; (**C**) benzoylhypaconine; (**D**) paeoniflorin; (**E**) isoliquiritigenin; (**F**) calycosin-7-glucoside; (**G**) tetrahydropalmatine; and (**H**) formononetin.

In the current study, various mobile phase solutions, including methanol, acetonitrile, ammonium acetate and formic acid, were tested to obtain optimized conditions including suitable retention times and good peak shapes for all analytes. Finally, a gradient elution with acetonitrile and 0.1% formic acid was chosen. With optimized precursor and product ions, the multiple reactions monitoring (MRM) mode was employed on a triple quadruple mass spectrometer, which provided high specificity for monitoring the target ion in a complex matrix, as shown in Figure 1. The typical chromatogram for detection of seven compounds in the stock solution and rat blank plasma were shown as the Supplementary Materials Figures S1 and S2. The mass spectrometer parameters, such as electrospray ionisation (ESI) source temperature, capillary and cone voltage, and flow rate of ion source gas, were also optimized to obtain the highest intensity of the analytes.

### 2.2. Method Validation

#### 2.2.1. Selectivity

The selectivity and specificity of this method was investigated by analyzing the chromatograms of six blank plasmas, spiked plasmas with standards and rat plasmas after oral administration of GJK. Under the established chromatographic condition, there were no significant interferences in the retention time of the drug or internal standard.

#### 2.2.2. Linearity and LLOQ

The regression equation, correlation coefficients and linearity ranges for the seven analytes are shown in Table 1. The linearity ranges were appropriate for the quantitative detection of the seven analytes in the pharmacokinetic studies. The calibration curves of the seven analytes exhibited good linearity with the coefficients of correlation (R^2^) better than 0.99. The lower limit of quantification (LLOQ) for benzoylhypaconine, benzoylmesaconine, paeoniflorin, tetrahydropalmatine, calycosin-7-glucoside, formononetin, and isoliquiritigenin were 0.335 ng/mL, 0.607 ng/mL, 11.3 ng/mL, 0.519 ng/mL, 0.619 ng/mL, 0.542 ng/mL, and 1.06 ng/mL, respectively. 

#### 2.2.3. Precision and Accuracy

The precision and accuracy data for the seven analytes in the quality control (QC) samples are presented in Table 2. The intra-day and inter-day precisions were 2.3%–18.0% and 1.7%–8.7%, respectively. The accuracy that is derived from the QC samples was between 92.9% and 107.8% for the three concentration levels of the seven analytes. All assay values were within the acceptable criteria, which indicated that the developed method has a satisfactory accuracy and precision.

#### 2.2.4. Extraction Recovery and Matrix Effect

The mean extraction recoveries of the seven components in plasma at three different concentration levels were found to be 85.4%–107.1% with RSD of less than 9.3% (Table 2), which indicated that sample processing was suitable for this method. The matrix effect values obtained for the analytes ranged from 86.8% to 102.4%, suggesting that the matrix effects for the analytes were negligible for this assay.

#### 2.2.5. Stability

The QC samples at three concentrations were used for stability evaluation of the seven analytes that were stored and processed under different conditions. The results of short-term stability, long-term stability, freeze-thaw stability and auto-sampler stability are summarized in Table 3, which showed that the differences between the measured values and the nominal values were within ±10.1%. Thus, the seven compounds were stable in rat plasma under these storage conditions. Therefore, a sensitive and reliable method by using UPLC–MS/MS analysis for simultaneous determination of seven constituents in rat plasma are successfully developed and validated.

### 2.3. Pharmacokinetic Study

**Figure 2:**
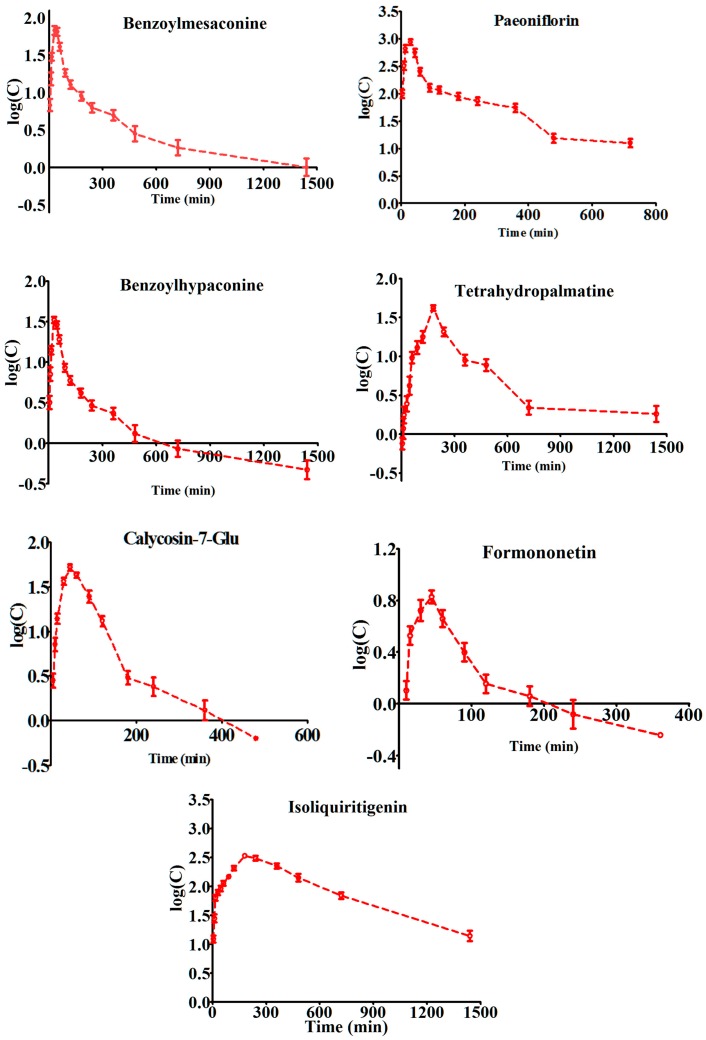
The (log) plasma concentration-time curves of seven compounds in rats after the oral administration of Guanjiekang 24 g/kg to rats (*n* = 6, mean ± SD).

This developed UPLC-MS/MS method was successfully applied to the pharmacokinetic study of seven compounds in rat plasma after a single oral administration of GJK. The mean plasma concentration–time profiles of the analytes are illustrated in Figure 2. The main pharmacokinetic parameters, including half-time (T_1/2_), maximum plasma concentration (C_max_), time to reach the maximum concentrations (T_max_), area under concentration-time curve (AUC_0__–t_ andAUC_0__–∞_), and mean resident time (MRT), were calculated and are listed in Table 4. As “non-compartmental” PK analysis was used in our study, an apparent terminal half-life is estimated from a subjective assessment of the concentration-time profile during an apparent mono-exponential phase. The time to reach the maximum plasma concentrations (T_max_) for benzoylhypaconine, benzoylmesaconine, paeoniflorin, calycosin-7-glucoside, and formononetin was less than 46 min, which indicates that most of these compounds are quickly absorbed into the blood circulation. However, the T_max_ values of tetrahydropalmatine and isoliquiritigenin were 189 min and 180 min, which indicates that they have a different absorption rate in vivo. Moreover, we found that C_max_ of the seven active compounds ranged from 6.79 to 905 ng/mL. In the recent in vitro studies, it has been found that paeoniflorin has antioxidative and anti-inflammatory activities with the half maximal inhibitory concentration IC_50_ values of 4.197 × 10^−4^ M (equal to 201.4 ng/mL) [26]. Furthermore, it inhibits the phosphorylation of extracellular signal-regulated kinase (ERK), c-Jun *N*-terminal kinases (JNK), IκB kinase (IKK) stimulated by tumor necrosis factor (TNF-α) at 25 ng/mL [27]. The in vivo concentration of paeoniflorin observed in this study is significantly higher than that of in vitro biologically effective doses, which indicates that it may be one of the active compounds for treating arthritis. Another active compound with a higher C_max_ at 337 ng/mL is isoliquiritigenin; it has recently been reported to block lipopolysaccharides (LPS)-induced toll-like receptor 4 (TLR4)/MD2 complex signaling and nuclear factor NF-κB activation [28] and to inhibit NACHT, LRR and PYD domains-containing protein 3 (NLRP3)-activated apoptosis-associated speck-like protein (ASC) oligomerization [29]. Interestingly, NLRP3-dependent interleukin (IL)-1β production has been inhibited with low concentrations of isoliquiritigenin in 1 µM (equal to 256 ng/mL) at both the priming step and the activation step that was below the C_max_ observed in our PK study [29]. Therefore, isoliquiritigenin could be another important active compound that contributes to the therapeutic effects of GJK. Further pharmacological studies for these seven compounds in combination with in vivo concentration would be interesting to provide the useful information for understanding the mechanism of herbal medicinal preparation. In summary, different constituents in GJK have different PK behaviors with diverse pharmacological effects that are produced at different time periods after oral administration in the clinic.

## 3. Materials and Methods

### 3.1. Herbal Materials and Chemicals

*Astragali Radix*, *Aconiti Lateralis Radix Praeparaia*, *Glycyrrhizae Radix et Rhizoma, Corydalis Rhizoma* and *Paeoniae Radix Alba* were collected from Good Agricultural Practice of Medicinal Plants in the provinces of Shanxi, Anhui, Gansu, Zhejiang and Sichuan. These five herbs were authenticated according to the Chinese Pharmacopoeia (edition 2015, volume 1) by Dr. Zhifeng Zhang (Macau University of Science and Technology), a professor in the herbal authentication research area. The voucher specimens were deposited in the State Key Laboratory (SKL) Quality Research in Chinese Medicine (Macau University of Science and Technology). Three batches of GJK preparation were prepared using a standardized protocol and quality control in SKL. Standard reference compounds of benzoylhypaconine, benzoylmesaconine, paeoniflorin, calycosin-7-glucoside, tetrahydropalmatine, isoliquiritigenin, formononetin and naringenin (internal standard, IS) were obtained from the National Institutes for Food and Drug Control (Beijing, China) with a purity of greater than 98%; formic acid was purchased from Sigma (St. Louis, MO, USA); acetonitrile and methanol (HPLC grade) were from Anaqua Chemicals Supply (Houston, TX, USA); distilled water was further purified by the Milli-Q system (Millipore, Milford, MA, USA); other chemicals were of analytical grade. All solvents and samples were filtered through 0.22 µM filters before injection into UPLC-MS.

### 3.2. Chromatographic and Mass Spectrometric Conditions

Chromatographic analyses were performed using an Agilent 1290 series UPLC system (Agilent Technologies, Santa Clara, CA, USA) equipped with a binary pump, an online degasser, an auto plate sampler, and a thermostatically controlled column compartment. The determination was carried out at 30 °C on a Waters Acquity UPLC C_18_ column (100 mm × 2.1 mm, 1.7 µM, Waters, Milford, MA, USA). The mobile phase consisted of 0.1% formic acid (A) and acetonitrile (B) using a gradient elution of 10%–34% B at 0–6 min, 34%–80% B at 6–10 min, 80% B at 10–12 min, 10% B at 12–15 min, and the re-equilibration time of gradient elution was 1 min. The sample injection volume was 5 µL, and the flow rate was 0.35 mL/min. The MS detection was performed using an Agilent 6460 Triple Quadrupole MS (Agilent Technologies) equipped with an electrospray ionization (ESI) source. Ionization of the seven compounds was achieved in the positive ESI mode. The MRM transitions included *m*/*z* 269.1→197.1 for formononetin, with a collision energy of 40 eV; *m*/*z* 447.1→285.1 for calycosin-7-glucoside, with a collision energy of 9 eV; *m*/*z* 590.3→105.1 for benzoylmesaconine, with a collision energy of 49 eV; *m*/*z* 574.3→105.1 for benzoylhypaconine, with a collision energy of 50 eV; *m*/*z* 257.1→137.1 for isoliquiritigenin, with a collision energy of 20 eV; *m*/*z* 356.2→192.1 for tetrahydropalmatine, with a collision energy of 25 eV; and *m*/*z* 498.2→179.1 for paeoniflorin, with a collision energy of 9 eV. Other parameters were as follows: drying gas (N_2_) flow rate, 11.0 L/min.

### 3.3. Preparation of GJK

Five herb plants, including *Astragali Radix* (6.5 kg), *Aconiti Lateralis Radix Praeparaia* (6.5 kg), *Glycyrrhizae Radix et Rhizoma* (3.9 kg), *Corydalis Rhizoma* (6.5 kg) and *Paeoniae Radix Alba* (7.8 kg), were extracted twice with distilled water (1:9 and 1:5, *v*/*w*, respectively) for 1.5 h and 1 h, respectively. The combined aqueous solution was concentrated under reduced pressure at 60 °C to prepare a concentrated liquid with a density of 1.08–1.12 g/cm^3^. Then, the extracts from the GJK herbal formula were sprayed to dry under a −0.5 KPa vacuum with an inlet temperature of 175 °C and an outlet temperature of 93 °C. The produced powder was stored at −20 °C. In the final product, the concentrations of benzoylhypaconine, benzoylmesaconine, paeoniflorin, tetrahydropalmatine, calycosin-7-glucoside, formononetin, and isoliquiritigenin were 85.4 µg/g, 359 µg/g, 1.50 × 10^4^ µg/g, 334 µg/g, 819 µg/g, 108 µg/g, and 134 µg/g, respectively. 

### 3.4. Preparation of the Stock, Standard and QC Solutions

The stock solutions of benzoylhypaconine, benzoylmesaconine, paeoniflorin, tetrahydropalmatine, calycosin-7-glucoside, formononetin, and isoliquiritigenin were prepared by dissolving the accurately weighed reference compounds in a water–methanol (50:50, *v/v*) solution. The standard working solutions were prepared via series dilution with water–methanol (50:50, *v/v*) to produce the final concentrations of 0.335–209 ng/mL for benzoylhypaconine, 0.607–379 ng/mL for benzoylmesaconine, 0.519–324 ng/mL for tetrahydropalmatine, 11.3–7.08 × 10^3^ ng/mL for paeoniflorin, 0.619–386 ng/mL for calycosin-7-glucoside, 0.542–338 ng/mL for formononetin, and 1.06–666 ng/mL for isoliquiritigenin. The IS solution (20 ng/mL) was prepared by dissolving a quantity of naringenin in methanol. Calibration samples were prepared by spiking 5 µL of working solutions at the corresponding concentrations into 50 µL of blank rat plasma. The LLOQ, and low, medium and high QC samples were prepared in the same way. All solutions were stored in a refrigerator (−40 °C) until analysis.

### 3.5. Plasma Sample Preparation

Plasma samples (50 µL) were thawed on ice and mixed with 200 µL of IS (20.0 ng/mL) via vortexing for 1 min. Then, the mixture was centrifuged at 13,500 rpm for 15 min to separate the precipitated protein at 4 °C. The supernatant was transferred into a clean Eppendorf tube and evaporated to dryness under a stream of nitrogen. The residues were reconstituted with 50 µL of water–methanol (50:50, *v*/*v*), vortexed and centrifuged at 13,500 rpm for 15 min. Then, the supernatants were injected into the UPLC–MS/MS for analysis.

### 3.6. Method Validation

The method was validated in terms of specificity, recovery, linearity, sensitivity (LLOQ), matrix effect, accuracy, precision and stability based on the USA Food and Drug Administration (FDA) bioanalytical method validation guidance.

#### 3.6.1. Specificity

The selectivity of the method was evaluated by analyzing blank plasma and plasma samples collected from six rats to investigate the potential interferences at the peak region of analytes and I.S. using the proposed extraction procedure and analytical conditions.

#### 3.6.2. Linearity and Low Limits of Quantification (LLOQ)

The linearity of each calibration curve was determined by plotting the peak area ratio of analytes to I.S. versus the nominal concentration of analytes with weighted (1/x^2^) least square linear regression. LLOQ was determined in accordance to the baseline noise, considering a signal-to-noise ratio of 10:1.

#### 3.6.3. Precision and Accuracy

The precision and accuracy were determined via the replicate analysis (*n* = 6) of QC samples at three concentration levels on the same day (intra-day) and on three different days (inter-day). The precision was calculated via relative standard deviation (RSD%), and the accuracy was expressed as (mean measured concentration/spiked concentration) ×100%. The data were acceptable if the precision (RSD%) was within 15% and the accuracy (%) was within ±15% of the nominal values.

#### 3.6.4. Extraction Recovery and the Matrix Effect

The extraction recovery was determined by comparing the mean peak areas of seven extracted samples at low, medium and high QC concentrations with the mean peak areas of the spike-after-extraction samples. The matrix effect was investigated by comparing the peak areas of analytes in the post-extraction spiked blank plasma at low, medium and high concentrations with those of the corresponding standard solutions. 

#### 3.6.5. Stability

The stability of the seven compounds in rat plasma was assessed by analyzing the QC samples at three concentrations under different sample storage conditions and processing procedures. Short-term stability was assessed by analyzing the QC samples that were kept at room temperature for 4 h, which exceeded the routine sample preparation time. Long-term stability was evaluated by keeping the QC samples at −40 °C for 15 days. Freeze-thaw stability was investigated after three freeze (−40 °C) and thaw (room temperature) cycles. Post-preparation stability was assessed by analyzing the extracted QC samples that were kept in the auto-sampler at 4 °C for 12 h. All stability testing QC samples were evaluated using the calibration curve of the freshly prepared samples. 

### 3.7. Pharmacokinetic Study

Sprague–Dawley rats (200–250 g) were obtained from the Center of the Chinese University of Hong Kong and were kept in an environmentally controlled room (temperature: 25 ± 2 °C, humidity: 50% ± 5%, 12 h dark-light cycle) for at least 7 days before the experiments. Prior to the experiments, the animals were fasted for 24 h with water ad libitum. All protocols of animal experiments were approved in accordance with the regulations of the Experimental Animal Administration issued by the Macau University of Science and Technology.

Pharmacokinetic study was carried out in rats after the oral administration (oral gavage) of the GJK extraction powder at a single dose of 24 g/kg/day, which has been demonstrated to have a significant anti-arthritis effect in animal models (data is not shown). The blood samples (100 µL) were individually collected from the caudal vein into heparinized tubes before administration, and 5, 10, 15, 30, 45, 60, 90, 120, 240, 360, 480, 720 and 1440 min after administration, and then immediately centrifuged at 4500 rpm for 15 min. The plasma samples were stored at −40 °C for later analysis of the concentration of benzoylhypaconine, benzoylmesaconine, paeoniflorin, tetrahydropalmatine, calycosin-7-glucoside, formononetin, isoliquiritigenin via the established UHPLC–MS/MS method. The main PK parameters, including the maximum plasma concentration (C_max_), time to reach C_max_ (T_max_), half-life time (T_1/2_), area under the curve (AUC _0–t_ and AUC _0–∞_) and mean residence time (MRT), were calculated via the non-compartment model using the pharmacokinetic software, PK Solutions 2.0 (Summit Co., Montrose, CO, USA).

## 4. Conclusions

In this study, a rapid, sensitive and specific UPLC–MS/MS method has been developed and validated for simultaneous determination of benzoylhypaconine, benzoylmesaconine, paeoniflorin, tetrahydropalmatine, calycosin-7-glucoside, formononetin, and isoliquiritigenin in rat plasma samples. The method was successfully applied to evaluate the PK profiles of seven constituents after the oral administration of Chinese herbal medicinal preparation GJK in rats. The PK behaviors of these seven compounds that are observed in this study indicated that these compounds can be quickly absorbed after the administration of GJK. Moreover, the in vivo concentrations of paeoniflorin and isoliquiritigenin are higher than the in vitro reported effective doses. This suggests that they are the major active compounds that can be used as markers for the quality control of herbal medicinal products as well as the monitoring targets in clinical studies.

## Figures and Tables

**Table 1:** Linear regression of the calibration curves of seven analytes in rat plasma.

Analytes	Linear Range (ng∙mL^−1^)	Regression Equation	R^2^
Benzoylhypaconine	0.335–209	*Y* = 0.00490*x* + 0.00200	0.9984
Benzoylmesaconine	0.607–379	*Y* = 0.00100*x* + 0.0237	0.9987
Paeoniflorin	11.3–7.08 × 10^3^	*Y* = 0.000500*x* + 0.0523	0.9977
Tetrahydropalmatine	0.519–324	Y = 0.0354*x* + 0.577	0.9936
Calycosin-7-glucoside	0.619–386	*Y* = 0.00760*x* − 0.163	0.9989
Formononetin	0.542–338	*Y* = 0.00290*x* + 0.00890	0.9991
Isoliquiritigenin	1.06–666	*Y* = 0.00220*x* − 0.00370	0.9938

**Table 2:** Precision, accuracy, recovery and matrix effect for seven analytes in rat plasma (*n* = 3).

Analytes	Concentration (ng/mL)	Inter-Day RSD (%)	Intra-Day RSD (%)	Accuracy	Recovery (%)	Matrix Effect (%)
Paeoniflorin	20.8	5.3	2.9	105.0%	86.1 ± 7.4	90.3 ± 3.4
	332	2.6	4.2	107.1%	90.8 ± 5.7	92.7 ± 3.1
	5315	4.7	1.8	106.8%	88.6 ± 3.4	90.9 ± 1.8
Tetrahydro-palmatine	0.949	4.7	2.1	101.9%	101.2 ± 6.9	101.6 ± 4.2
	15.2	8.9	4.8	102.5%	90.4 ± 3.7	105.3 ± 3.6
	243	5.6	3.6	96.3%	85.8 ± 3.7	95.3 ± 2.5
Benzoylmes-aconitine	1.11	5.8	3.8	94.1%	95.1 ± 5.8	93.4 ± 5.6
	17.8	4.8	8.6	107.8%	96.7 ± 3.9	94.2 ± 5.2
	285	3.9	5.2	103.4%	94.6 ± 2.4	87.8 ± 3.1
Calycosin-7-glucoside	1.13	4.5	8.3	106.7%	93.6 ± 9.3	86.8 ± 2.0
	18.1	3.8	2.6	103.6%	90.6 ± 7.2	102.4 ± 3.6
	290	2.3	2.3	102.5%	86.6 ± 6.1	92.5 ± 2.7
Benzoylhyp-aconitine	0.614	9.7	2.7	92.9%	107.0 ± 7.8	95.3 ± 7.2
	9.84	6.3	6.9	105.8%	89.7 ± 5.2	98.2 ± 6.4
	157	6.4	4.2	104.6%	93.5 ± 4.1	87.2 ± 5.4
Isoliquiritigenin	1.95	2.7	8.7	103.5%	101.4 ± 4.9	93.8 ± 7.2
	31.2	4.5	4.3	103.1%	90.4 ± 3.7	98.2 ± 6.0
	499	18	3.7	102.1%	85.4 ± 4.2	93.1 ± 4.1
Formononetin	0.993	5.8	6.4	107.2%	87.2 ± 6.8	96.5 ± 8.2
	15.9	4.5	6.1	103.9%	91.3 ± 3.4	88.6 ± 7.1
	254	2.5	1.7	102.3%	86.7 ± 2.5	91.2 ± 3.8

RSD (%): Relative standard deviation.

**Table 3:** Stability of seven analytes in rat plasma under different storage conditions (*n* = 3).

Analytes	Conc. ng/mL	25 °C for 4 h		At −70 °C for 15 Days		Freeze–Thaw Cycles		4 °C for 24 h	
		RSD (%)	Remaining (%)	RSD (%)	Remaining (%)	RSD (%)	Remaining (%)	RSD (%)	Remaining (%)
Paeoniflorin	20.8	5.4	101.5	4.5	98.6	5.6	107.8	6.1	98.3
	332	3.2	102.3	5.2	103.6	6.2	106.4	2.5	105.4
	5315	2.8	96.4	2.3	104.0	3.9	105.0	1.3	103.2
Tetrahydro-palmatine	0.95	8.9	102.7	8.4	103.6	9.2	89.9	4.6	105.2
	15.2	6.4	101.1	3.7	102.7	8.5	107.4	7.2	98.4
	243	6.5	102.5	1.5	103.1	6.0	108.1	3.1	101.1
Benzoylmes-aconine	1.11	7.1	94.4	2.3	93.2	4.6	93.3	5.2	110
	17.8	4.8	107.1	4.9	105.4	5.3	104.8	6.7	96.3
	284	3.2	104.2	3.6	102.6	4.1	104.2	4.2	104.2
Calycosin-7-glucoside	1.13	8.5	105.9	6.8	108.4	9.9	108.1	4.8	103.7
	18.1	7.6	96.0	4.3	94.3	6.4	107.3	1.7	103.9
	290	6.3	103.2	2.8	102.2	7.1	107.4	2.7	102.8
Benzoylhyp-aconine	0.61	9.8	108.3	7.4	107.8	8.5	109.6	8.9	106.7
	9.83	7.6	107.6	8.6	104.3	6.7	92.7	5.4	104.3
	157	6.2	105.1	2.3	105.2	7.0	106.9	2.6	103.8
Isoliquiritigenin	1.95	5.2	102.4	5.6	103.1	5.6	106.3	2.8	94.6
	31.2	3.4	103.0	7.1	96.2	5.9	107.0	1.7	102.5
	499	4.1	102.6	4.6	101.6	3.2	105.2	3.9	102.1
Formononetin	0.99	5.8	96.9	6.9	103.5	6.7	104.8	6.4	93.3
	15.8	2.3	104.6	3.1	104.8	8.3	93.3	6.3	105.9
	254	3.7	103.4	4.8	104.3	5.6	105.0	5.4	106.2

RSD (%): Relative standard deviation.

**Table 4:** Pharmacokinetic parameters of seven major compounds after the oral administration of Guanjiekang in rats (*n* = 6).

Parameters	T_1/2z_ (min)	C_max_ (ng/mL)	T_max_ (min)	AUC_0–t_ (ng∙min/mL)	AUC_0–∞_ (ng∙min/mL)	MRT_0–t_ (min)
Paeoniflorin	210 ± 38	905 ± 226	30 (15, 30)	73,270 ± 22,823	73,749 ± 25,492	199 ± 31
Tetrahydropalmatine	310 ± 54	42.0 ± 13.4	180 (180, 180)	10,193 ± 2945	11,028 ± 3125	492 ± 63
Benzoylmesaconitine	687 ± 96	71.6 ± 21.5	30 (30, 45)	7998 ± 2159	9030 ± 2356	515 ± 48
Calycosin-7-glucoside	266 ± 44	52.2 ± 14.8	45 (45, 60)	4643 ± 1485	4759 ± 1793	130 ± 24
Benzoylhypaconine	382 ± 65	35.6 ± 10.2	30 (30, 45)	4327 ± 1341	4603 ± 1583	375 ± 43
Isoliquiritigenin	293 ± 48	337 ± 103	180 (180, 240)	159,115 ± 44,552	165,128 ± 46,094	465 ± 52
Formononetin	951 ± 97	6.79 ± 2.18	45 (30, 45)	912 ± 264	1123 ± 324	777 ± 64

T_1/2z_: terminal elimination half-life; C_max_: maximum plasma concentration; T_max_: the median value of time to reach peak concentration (minimum and maximum values); AUC_0–t_: area under the curve from zero to the last sampling time; AUC_0–∞_: AUC_0–t_ extrapolated to infinity; MRT_0–t_: mean residence time. Data were presented as mean ± S.D.

## Supplementary Materials

Supplementary materials can be accessed at: http://www.mdpi.com/1420-3049/21/12/1732/s1.
