# Supplementary material for: Simultaneous Determination of Multiple Components in Guanjiekang in Rat Plasma via the UPLC–MS/MS Method and Its Application in Pharmacokinetic Study
Source: Molecules. 2016 Dec 16;21(12):1732. doi: 10.3390/molecules21121732 (PMC6272869; doi:10.3390/molecules21121732)
Supplement: Supplementary file 1 [file molecules-21-01732-s001.pdf]

Supplementary Materials: Simultaneous Determination of Multiple Components in Guanjiekang in Rat Plasma via the UPLC–MS/MS Method and Its Application in Pharmacokinetic Study

Jian Wu, Ying Xie, Zheng Xiang, CanJian Wang, Hua Zhou and Liang Liu

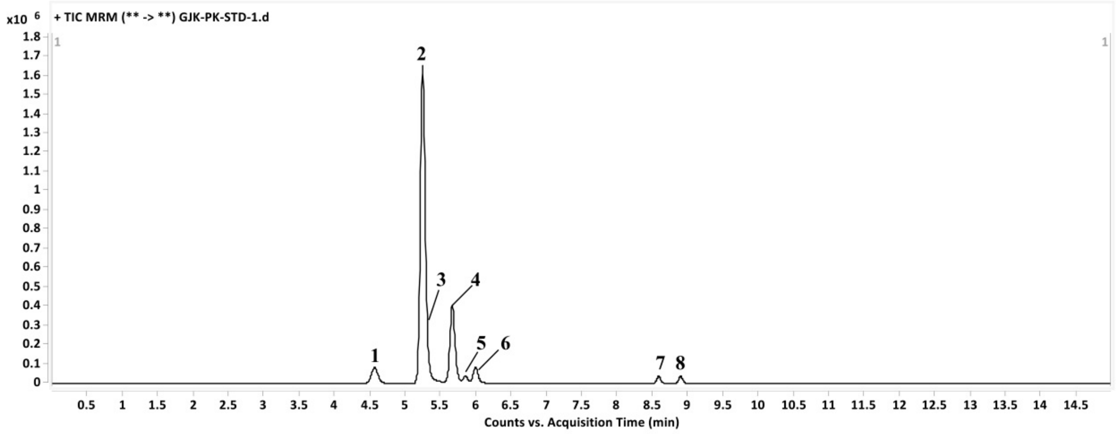

Figure S1. The chromatogram of stock solution. (1: Paeoniflorin, 2: Tetrahydropalmatine, 3: Benzoylmesaconine, 4: Calycosin-7-glucoside, 5: Benzoylhypaconine, 6: Isoliquiritigenin, 7: Naringin, 8: Formononetin).

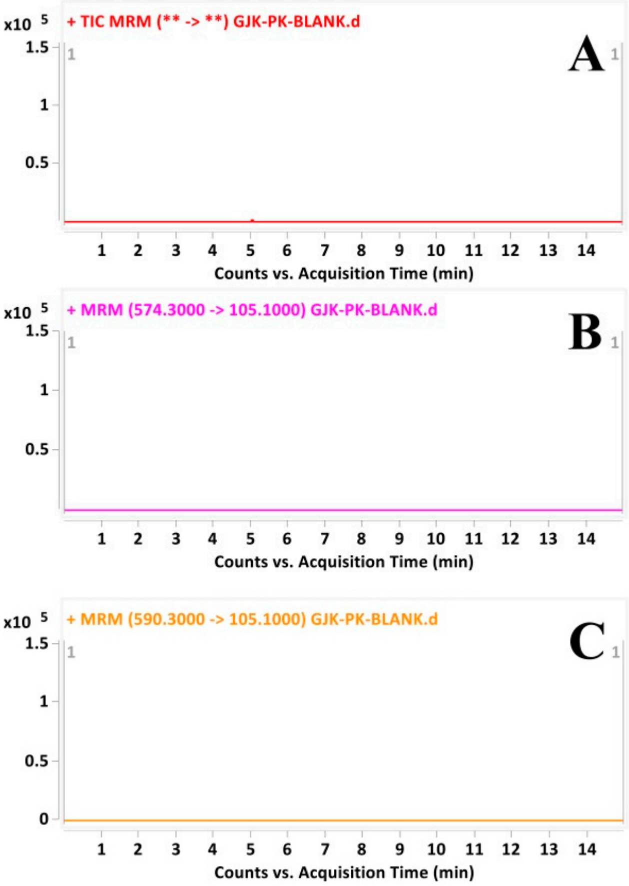

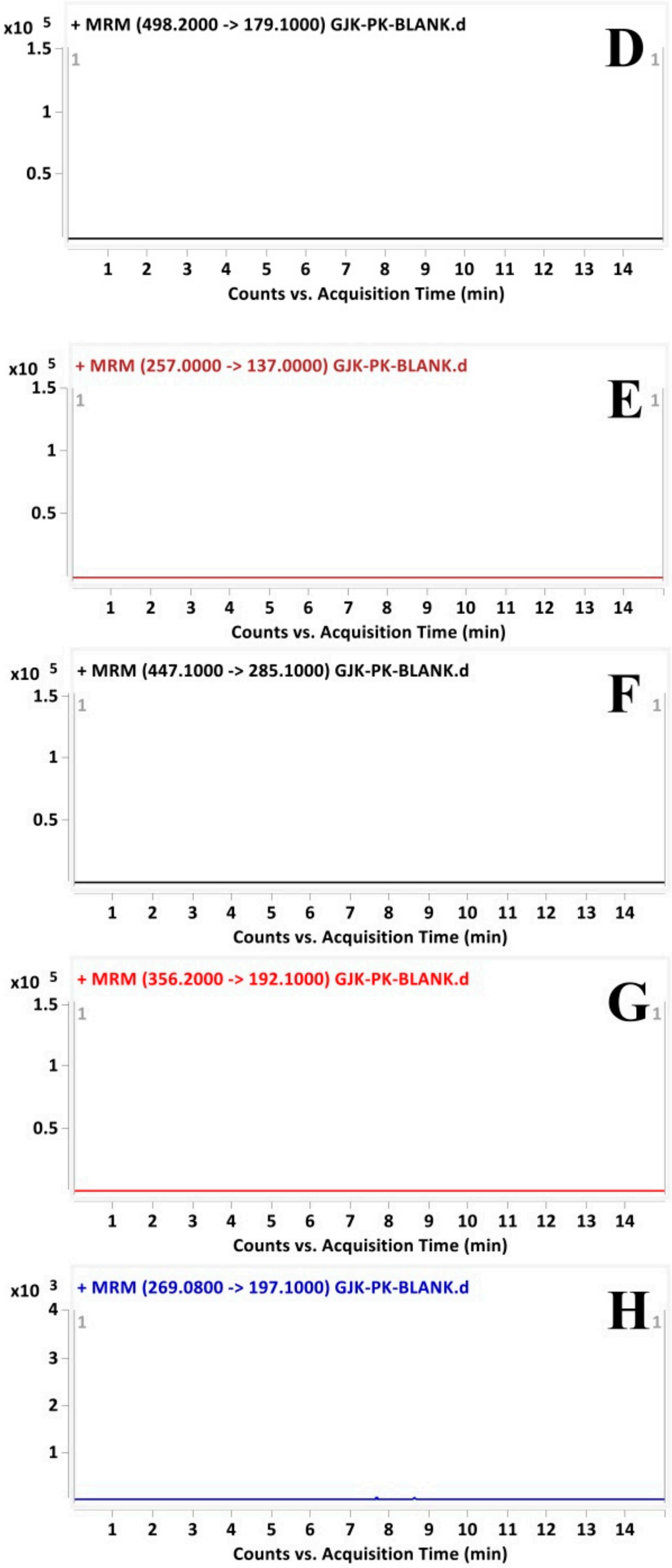

Figure S2. The chromatogram of rat blank plasma. ((**A**): blank of rat plasma; (**B**): determination of benzoylmesaconine in blank plasma; (**C**): determination of benzoylhypaconine in blank plasma; (**D**): determination of paeoniflorin in blank plasma; (**E**): determination of isoliquiritigenin in blank plasma; (**F**): determination of calycosin-7-glucoside in blank plasma; (**G**): determination of tetrahydropalmatine in blank plasma; (**H**): determination of formononetin in blank plasma).
